# Focused peptide library screening as a route to a superior affinity ligand for antibody purification

**DOI:** 10.1038/s41598-021-91208-0

**Published:** 2021-06-02

**Authors:** Krištof Bozovičar, Barbara Jenko Bizjan, Anže Meden, Jernej Kovač, Tomaž Bratkovič

**Affiliations:** 1 Faculty of Pharmacy, Department of Pharmaceutical Biology, University of Ljubljana, Aškerčeva 7, 1000 Ljubljana, Slovenia; 2 Clinical Institute of Special Laboratory Diagnostics, University Medical Center, University Children’s Hospital, Vrazov trg 1, Ljubljana, Slovenia; 3 Faculty of Pharmacy, Department of Pharmaceutical Chemistry, University of Ljubljana, Aškerčeva 7, 1000 Ljubljana, Slovenia

**Keywords:** Biochemistry, Biophysics, Biotechnology, Chemical biology

## Abstract

Affinity chromatography is the linchpin of antibody downstream processing and typically relies on bacterial immunoglobulin (Ig)-binding proteins, epitomized by staphylococcal protein A-based ligands. However, such affinity ligands are fairly costly and suffer from chemical instability, leading to ligand denaturation and leaching from chromatographic support. Innovations in this area are aimed at developing robust and highly selective antibody ligands capable of withstanding harsh column sanitization conditions. We report the development and first-stage characterization of a selective short linear peptide ligand of the IgG Fc region capable of capturing all four IgG subclasses. The ligand was discovered through in vitro directed evolution. A focused phage-display library based on a previously identified peptide lead was subjected to a single-round screen against a pool of human IgG. The hits were identified with next-generation sequencing and ranked according to the enrichment ratio relative to their frequency in the pre-screened library. The top enriched peptide GSYWYNVWF displaying highest affinity for IgG was coupled to bromohydrin-activated agarose beads via a branched linker. The resulting affinity matrix was characterized with a dynamic binding capacity of approx. 43 mg/mL, on par with commercially employed protein A-based resin.

## Introduction

Isotype G immunoglobulins (IgGs) are widely used as biorecognition reagents in basic molecular biology research and diagnostics^1^, including enzyme-linked immunosorbent assay (ELISA), western blot, immunocytochemistry, fluorescence-activated cell sorting, immunoprecipitation, and for selective capture of analytes in biosensor detection/quantification. They are distinguished by high affinity, owing to the preorganized conformation of highly specific complementarity-determining region loops that interact with an antigen. The invariant portion of IgGs, specifically the Fc region, enables convenient use of labeled isotype-specific secondary antibodies as universal detection agents. In addition, fusion proteins containing IgG Fc region have become popular research tools^2^ due to dimeric display of targeting moiety (and hence avidity effects), generic purification schemes, universal detection, and long in vivo half-life as a result of neonatal Fc receptor-mediated recycling in endothelial cells.

Platforms for development, maturation, and production of monoclonal antibodies (mAbs) are well-established. mAbs are developed by immunization of animals (typically mice) followed by hybridoma technology to gain access to IgG-producing cell clones or via phage display technology^1^. The complex structure and post-translational modifications necessitate costly mAb expression in mammalian cells. In recent years, mammalian cells were engineered to secrete large amounts of antibodies and Fc-fusion proteins, and it is the downstream processing that accounts for the majority of production costs^3^. Affinity chromatography based on bacterial immunoglobulin-binding proteins (i.e., staphylococcal protein A (spA) or streptococcal protein G (spG)) represents the cornerstone of antibody isolation and purification, allowing high clearance of process-related impurities, and concomitant antibody product concentration^4^. Such protein affinity ligands are by no means ideal^5^. Firstly*,* they form high-affinity interactions with the antibody Fc region, requiring harsh conditions for IgG elution—these, in turn, can shorten the affinity column lifetime due to ligand denaturation, and can negatively affect product structural and functional integrity (resulting from antibody denaturation and aggregation). Secondly, they themselves are produced by recombinant DNA technology, translating to high affinity column costs. Finally, ligand leaching as a result of inter-run column sanitation (based on washing with highly alkaline solutions) leads to column deterioration, while proteolysis exacerbated by concomitant introduction of proteases with the feedstock requires extensive efforts to eliminate immunogenic ligand fragments, especially if the antibody product is to be used in vivo. Thus, there is high demand for alternative ligands for affinity capture of IgG with improved properties.

Short peptides and analogs thereof represent a viable alternative to complex protein ligands as biorecognition reagents^6^. Despite being small in size (and thus available through cost-efficient chemical synthesis with consistent quality even on a large scale), peptides engage in specific interactions with proteins. Importantly, peptides are very stable (denaturant-resistant) compared to larger proteins that rely on complex folding patterns to form binding sites. Peptides as affinity ligands for chromatography additionally have the advantage of binding to target proteins with moderate affinity, thus requiring mild elution conditions, preserving the binding partner’s structural integrity, while the resin’s binding capacity can be efficiently improved through modulation of linker length, rigidity, and ligand-coupling density. Furthermore, the potential leaching of peptides from affinity matrices is not a major issue regarding the augmented product immunogenicity, and can be minimized by engineering protease-resistant features in peptides, such as incorporation of non-natural amino acids^7^ or replacement of the peptide backbone with a nonpeptide scaffold^8,9^.

In our previous work^10^, we reported the identification and structure–activity relationship analysis of a short linear peptide ligand binding to human IgGs. The lead peptide was identified by screening a random dodecapeptide combinatorial phage-display library against the pool of human IgG Fc. Analog peptides were designed by N- and/or C-terminal trimming, residue substitutions (alanine scanning, conservative or non-conservative replacements), and deletions, and their affinity was comparatively evaluated with respect to the parent peptide in phage ELISA assays. Coupled to paramagnetic beads or cross-linked agarose matrix, the optimized peptide ligand min19Fc-Q6D (GSYWYDVWF) was shown to selectively enrich antibodies from complex protein mixtures in pull-down assays and affinity chromatography.

Here, we describe the development of an optimized peptide ligand and its application in affinity chromatography of antibodies. We designed a secondary (i.e., focused) phage display peptide library based on randomization of selected non-essential residues of parent peptide min19Fc-Q6D^10^ and screened it against the human IgG pool. A single-step panning was conducted, followed by deep sequencing of the retained phage clones, to avoid growth bias that can occur between selection cycles and to quantitatively assess hit enrichment. A prototype affinity column based on the improved peptide ligand variant was constructed and characterized in comparison to those based on the parent peptide min19Fc-Q6D or the conventional spA ligand. We show that the optimized peptide-functionalized affinity matrix displays dynamic binding capacity that is comparable to the spA-based commercial one, and facilitates isolation of human IgGs of all four subclasses. The bound IgGs can be quantitatively eluted at mild conditions, while the affinity matrix is highly resistant to sodium hydroxide solutions used for column sanitization.

## Methods

### Peptide library construction and screening

Phagemid pIT2 was modified to remove the encoded long peptide linker (i.e., AAA–his-tag–GAA–myc-tag–GAAQ) connecting the displayed polypeptide and p3 phage minor coat protein, leaving only the short trialanyl spacer. The plasmid backbone was amplified with Q5 High-Fidelity Polymerase (New England Biolabs) using primers F-SL (5′-aaaa*GCGGCCGC*AACTGTTGAAAGTTGTTTAGCAAAACCTCATACAGAAAATTCATTTACTAACG-3′) and R-SL (5′-aaaa*CCATGG*CCGGCTGGGC-3′; vector-complementary sequences are underlined, lower case letters denote arbitrary nucleotides, and the *Not*I and *Nco*I restriction sites, respectively, are italicized). The ~ 4.5 kb amplicon was gel purified and double digested with *Not*I/*Nco*I. Complementary stuffer oligonucleotides F-stuffer (5′-*CATGG*CC**TAATAATAA**AAGACGGACAACTAGGTA*GC*-3′) and R-stuffer (5′-*GGCCGC*TACCTAGTTGTCCGTCTT**TTATTATTA**GG*C*-3′; stop codons denoted in bold) were dissolved in water, mixed in an equimolar ratio (50 µM each), heat-denatured at 98 °C, and slowly cooled to room temperature to anneal. The mixture was diluted in a 1:125 ratio, and 1 µL was used as an input for ligation reaction together with 50 ng of pIT-2 backbone amplicon. The resulting phagemid vector termed pIT2-SL (SL stands for short linker) was amplified in *E. coli* TOP10 bacteria and sequenced.

To construct the phage display library of IgG-binding peptide variants with the anticipated diversity of 64,000 clones, 5 µg of the degenerate library oligonucleotide (5′-aatt*CCATGG*CCGGTNNKTWTTGGTWT**NNN**NNKTGGTWT*GCGGCCGC*ctaacgtaacgaccag-3′, where N denotes any nucleotide, K is G or T, W is A or T, while the softly randomized codon ([10% A/10% C/ 70% G/10% T][70% A/10% C/10% G/10% T][10% A/10% C/10% G/70% T]) is depicted in bold) was annealed with the extension oligonucleotide (5'-ctggtcgttacgttag*GCG*-3'; both from GenScript) at 1:3 molar ratio as described above, and extended with Klenow fragment in NEBuffer 2 (New England Biolabs). The double-stranded product was phenol/chloroform-extracted and concentrated by ethanol precipitation before being digested with *Nco*I/*Not*I and gel purified. Finally, the degenerate library insert was subcloned into linearized pIT2-SL and used to transform chemically competent *E. coli* TG1 cells. The bacteria were streaked on 15 cm Petri plates with 2 × TY agar medium supplemented with 1% glucose and 100 µg/mL ampicillin, and the colonies were collected by gentle scraping in 2 × TY/1% glucose medium. Library phagemids were rescued by infecting transformed bacteria with KM13 helper phage, virions were concentrated with PEG/NaCl precipitation and suspended in PBS.

Phage library was screened against the MaxiSorp (Thermo Scientific) surface immunotube-immobilized polyclonal human IgG (Octagam, Octapharma), applying sequential elution using buffers of progressively descending pH values (50 mM citrate–phosphate pH 5.6, 4.6, and 3.6; and 200 mM glycine–HCl pH 2.2). The library was diluted in 800 µL PBS buffer, pH 7.4, contacted with the target for 1 h, and washed 10 times with 4 mL 0.1% PBST. After every elution step (8 min incubation each), the tubes were rinsed three times with 4 mL of the same elution buffer before proceeding with the next elution step utilizing more stringent conditions. Alternatively, phages were eluted in a single step with 800 µL of 100 mM Tris buffer pH 9.0. Two parallel single-round panning experiments were performed. The neutralized eluted phage fractions were used to transduce *E. coli* TG1 host strain and plated. Colonies were scraped in bacterial medium and harvested by centrifugation. Finally, pooled phagemids were extracted from bacterial pellets with the Gene Elute Plasmid Miniprep kit (Sigma) and subjected to massive parallel sequencing.

### Sequencing of phagemid pools and hit ranking

Library phagemids and those collected from eluted fractions during library screening were used to prepare short reads NGS library, following the standard protocol and quality recommendations by Illumina (Nextera DNA Flex Library Preparation Kit, Illumina). In short, tagmentation and adapter ligation were performed, followed by amplification and indexed adapter ligation to purified tagmented DNA. Finally, libraries were quantified with Agilent Technologies Bioanalyser, pooled and prepared for paired-end sequencing on Illumina MiSeq.

Raw reads were sorted according to the specified barcodes and distributed to separate files. Sequences from the initial plasmid library were used to construct plasmid assembly. Low-quality reads and adapter sequences were trimmed using Prinseq^11^. Resulting sequences were normalized with BBNorm^12^ to achieve flat coverage distribution of reads. SPAdes-3.12.0-Linux^13^ was used to construct phagemid assembly from trimmed and normalized reads of initial phagemid library. Reads from eluted fractions were aligned to the assembled initial sequence using bwa mem aligner^14^. Next, for each eluted fraction, only reads spanning inserted sequence were selected for further analysis. Resulting sequences in bam file format were converted to fastq format (using bedtools bamtofastq) in order to trim for sequencing adapters and select reads spanning the whole insert sequence (27 base pairs) using cutadapt^15^.

Finally, the codon (residue) frequencies per each randomized position in resulting fragments were analyzed and calculated relative to the residue frequencies in initial library. Similarly, enrichment of individual peptides was assessed relative to their proportion in the pre-screened library. Data from two independent screening experiments were compared to verify the enrichment factors. For all statistical analysis R programming language^16^ and packages Biostrings, seqinr, and TraMineR were used.

### Phage ELISA assays

Top 12 screening hits and the parent peptide min19Fc-Q6D were displayed on phagemid virions and their relative affinities to human polyclonal IgG were compared in quantitative phage ELISA assay. Cognate codon-optimized complementary oligonucleotide pairs encoding individual peptides were annealed and subcloned in pIT2-SL phagemid as described above, and the virions were rescued by superinfection with KM13 helper phage. Upon isolation, phage clone concentration was determined spectrophotometrically^10^. Initially, 2 × 10^9^ virions per microtiter well were subjected to phage ELISA to assess the peptides’ relative affinities to human IgG pool. Additionally, for the 5 best binders ELISA was conducted with serial phage dilutions (ranging from 2.5 × 10^10^ to 1.95 × 10^8^ virions per well). The experiments were performed in triplicate using a MaxiSorp microtiter plate coated with IgG (Octagam at 5 µg/mL) and blocked with skimmed milk. After washing the wells, phages were detected with HRP-conjugated anti-M13 monoclonal antibody (GE Healthcare) and TMB substrate.

### Peptide coupling to cross-linked agarose

The choice of resin, linker, and coupling strategy was adopted from Islam et al.^17^ with the following modifications. Briefly, WorkBeads 40/1000 ACT resin (50% slurry with the nominal capacity of 200 µmol bromohydrin groups/mL) was incubated with 10 molar equivalents of neat tris(2-aminoethyl)amine overnight. The aminated resin was then thoroughly washed with 10 volume equivalents of 20% ethanol, followed by 10 volumes of dimethylformamide (DMF). *N*-Ethyl-*N*′-(3-dimethylaminopropyl)carbodiimide hydrochloride (5 eq.), *N*-hydroxyphthalimide (5 mol. eq.), and bromoacetic acid (5 mol. eq.) were dissolved in DMF (3 mL/mL resin) and stirred at room temperature for 15 min. The resulting solution was then added to the aminated resin, followed by diisopropylethylamine (DIPEA) (10 mol. eq.), and the resultant dark orange suspension was shaken at room temperature for 6 h. Afterward, the suspension was filtered on a glass frit and washed thoroughly with 20 volume equivalents of DMF. No residual free amine groups were present in the resin, as indicated by the negative Kaiser test. The Cys-terminated synthetic peptides min19Fc-Q6D (GSYWYDVWFC-CONH_2_) or peptide A (GSYWYNVWFC-CONH_2_; both > 90% pure as confirmed by HPLC and MS analysis; GenScript) were dissolved (5 mg/mL) in DMF/DIPEA = 9:1 (*v*/*v*) and DMSO/DIPEA = 9:1 (*v*/*v*), respectively, added to the bromoacetylated resin (40 mg peptide/mL resin), and shaken overnight. Neat 2-mercaptoethanol (20 molar eq.) was then added, and the suspension was shaken for further 6 h to block the unreacted residual bromoacetyl groups on the resin. The resin was then filtered on a glass frit, thoroughly washed—first with 20 volume equivalents of the coupling solvent (DMF or DMSO, respectively), followed by 10 volume equivalents of 20% ethanol—and stored in 20% ethanol at 4 °C.

### Characterization of peptide-based affinity column

#### Dynamic binding capacity assessment

Each peptide-coupled resin was packed into G-Trap 1 mL FPLC column (G-Biosciences). For a side-by-side comparison, protein A-based column BabyBio A (Bio-Works) was used. 0.01% PBST pH 7.4 was used as running/washing buffer, whereas 20 mM glycine∙HCl pH 3.0 and 100 mM Na-citrate pH 3.0 were used as elution buffers for peptide-coupled resins and BabyBio A, respectively. All columns were pre-equilibrated with the running buffer. 1 mg/mL IgG1 monoclonal antibody infliximab (Remsima, Celltrion) in the running buffer was used for dynamic binding capacity (DBC) determination. IgG1 was loaded onto columns at 0.5 mL/min flow rate. Washing and elution steps were performed at 1 mL/min and 0.5 mL/min, respectively. DBC was determined at 10% breakthrough. For peptide A and min19Fc-Q6D columns, cleaning-in-place (CIP) with 5 mL 0.5 M NaOH was performed between all runs at 2 mL/min. A long contact time (4 h) in 0.5 M NaOH with intermediate holds of 20 min was also performed between two consecutive DBC assessments for peptide A-based affinity column.

#### Assessment of binding different IgG subclasses

Binding of monoclonal antibodies panitumumab (IgG2; Vectibix, Amgen), nivolumab (IgG4; Opdivo, Bristol-Myers Squibb), and IgG3 from human plasma (Athens Research & Technology) to the peptide A resin was assessed. 10 mL of panitumumab (0.51 mg/mL) and nivolumab (0.62 mg/mL) were loaded, while 0.8 mL of IgG3 (1.3 mg/mL) were injected onto the column in running buffer. Flow rates were the same as for DBC determination.

#### Purification of IgG1 from mixture with bovine serum albumin

Infliximab (IgG1, 1 mg/mL in the running buffer) was supplemented with 4 mg/mL BSA and 10 mL portions were loaded onto all three columns. Flow rates were the same as for the DBC determination. Flow-through and eluted fractions were collected, and 10 µL of each were analyzed by SDS-PAGE to determine separation efficiency.

## Results

### Library construction and screening

**Figure 1:**
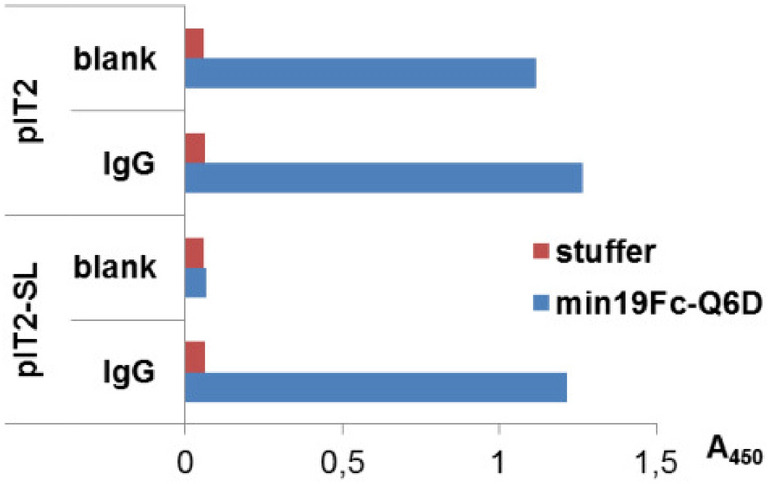
Binding of pIT2 and pIT2-SL min19Fc-Q6D-displaying virions to human IgG-coated and blank wells (skimmed milk-blocked only) in phage ELISA assay. Wild-type phenotype virions (with stuffer insert harboring stop codons on pIT2 and pIT2-SL background) were used as controls.

In affinity maturation campaigns involving variant library screening, avidity effects should be avoided as they interfere with enrichment of optimal binders. We thus resorted to a phagemid-based library wherein each peptide is expressed on the phage coat in at most one copy. However, when the parent peptide min19Fc-Q6D was displayed on pIT2 phagemid virion, there was substantial non-specific binding to blank (skimmed milk-blocked) microtiter wells in phage ELISA, as well (Fig. 1). We speculated that this might be due to the extremely long linker anchoring the displayed peptide to the p3 phage coat protein, potentially resulting in displayed hydrophobic peptide-linker fusion forming a random coil. Indeed, when we shortened the 27-residue linker to a tripeptide, the background signal dropped considerably, and highly selective binding to human IgG was observed (Fig. 1). We have thus confirmed that the peptide ligand is accessible for binding to IgG when displayed on a short linker, and that the parent peptide’s affinity is high enough to allow the binding of phage virions to antibodies in the monovalent display setting, supporting construction of a focused variant library in the modified pIT2-SL phagemid.

Based on preliminary low-throughput sequence space exploration of the parent peptide structure by on-phage characterization of variants in ELISA assay^10,18^, we noted that the general architecture of our peptides tolerates numerous structural modifications without the loss of binding affinity for IgG, implying that further improvements with respect to binding strength and/or type of bonding interactions (important for optimizing conditions used for peptide: antibody complex dissociation) are possible. To systematically interrogate the peptide’s sequence space, we constructed a focused library in which the residues found essential for Fc region binding were kept unchanged (W4 and W8; no substitutions were tolerated at these positions) or only conservative substitutions in equal ratios were allowed (i.e., Y3F, Y5F, and Y9F). Conversely, positions occupied by the more ‘promiscuous’ residues were either fully (S2 and V7) or partially (‘softly’) randomized (D6; substitution of the original Q with D at this particular residue was deemed favorable with respect to affinity towards Fc^10^). For ‘hard’ randomization, NNK codons were used^19,20^. This strategy allows for inclusion of all 20 amino acids while minimizing degeneracy in the third position of each codon. Reduced codon redundancy results in a more uniform distribution of amino acids within a peptide sequence and significantly reduces the frequency of terminations (only one of the three stop codons is allowed, and this can be counteracted by an amber suppressor *E. coli* host strain such as TG1). For soft randomization at position 6, we chose to favor the aspartate residue but allow incorporation of other amino acids as well. This was achieved by fixing the quantitative ratios of nucleotides at particular position of the codon (to 70% G, 70% A, and 70% T at nucleotide positions 1, 2, and 3, respectively, while the remaining three nucleotides were fixed at 10% each), meaning that the expected frequency of aspartate at position 6 was 39.2%, while those of other amino acid residues ranged from 0.1% (methionine and tryptophan) to 9.8% (glutamate). Position 1 was invariantly occupied by glycine, chosen as an inert residue to ensure equal cleavage of the leader sequence from all displayed peptides, since the signal peptidase might exhibit different activity at P1’ site when occupied by different residues. Glycine at this position was found to be well tolerated in the previous study^10^ with regard to IgG affinity. The degenerate library insert was subcloned into pIT2-SL phagemid and used to transform the TG1 bacterial host strain. The pIT2-SL vector harbors a stuffer with 3 consecutive *ochre* stop codons. Thus, any phage clones resulting from back-ligation (i.e., those missing the library insert) mimic the wild-type filamentous phage phenotype and should not interfere with affinity selection.

**Figure 2:**
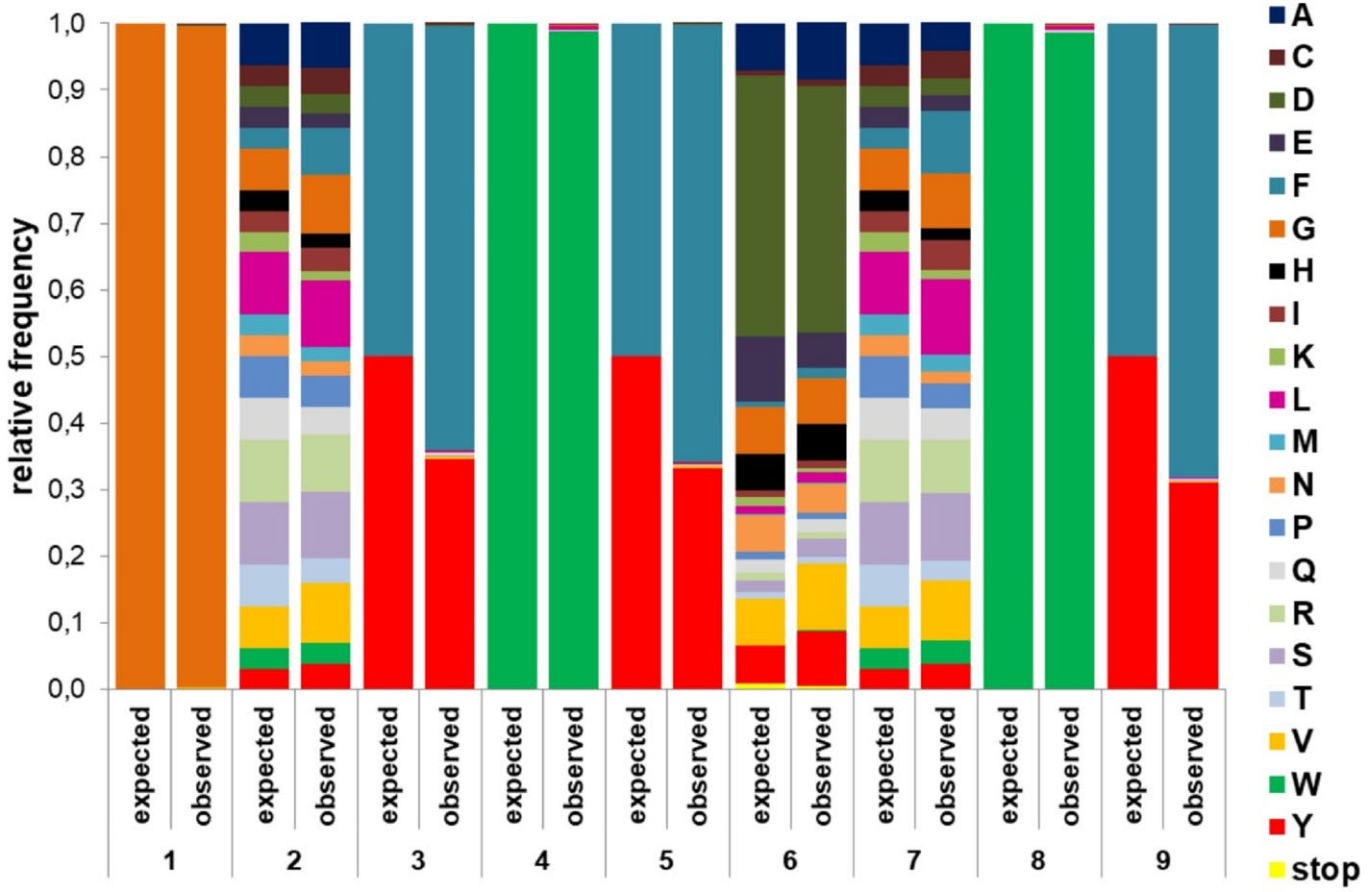
Relative expected and observed frequencies of amino acid residues at individual peptide positions of the pre-screened phage library.

A small aliquot of library-transformed bacteria was subjected to microbiological tittering, estimating the number of independent transformants at approx. 200,000, roughly three times that of the expected library diversity (64,000 peptides). Prior to panning experiments, the library was sequenced to verify that the observed residue frequencies at individual positions match the expected ones (Fig. 2). There was a good agreement between the two, except for positions 3, 5, and 9, which were biased towards phenylalanine (with Phe:Tyr ratio of ≈ 2:1 instead of expected 1:1).

In conventional phage library screening, several selection rounds are performed wherein phage are amplified in host bacteria following each panning. This can, however, bias repertoire diversity due to the growth advantage of individual clones^21^. By limiting screening campaigns to a single round and taking advantage of deep sequencing, this problem is avoided. Another common problem in phage display is that a significant proportion of phage particles might be carried over to the next selection round non-specifically (representing background). To identify specifically retained clones, 2 independent single round screening experiments were performed in parallel. The sequences obtained were compared and identical peptides enriched in both experiments were considered true hits^22^. Such analysis, not possible with conventional clone picking approach at the end of the screening campaign as it requires sequencing of large numbers of clones, greatly facilitates objective hit selection.

To secure enrichment of high-affinity binders, a large excess of library phages (3.8 × 10^13^) was allowed to compete for a limited number of adsorbed target molecules. Furthermore, we performed a stepwise elution using buffers of progressively descending pH values in an attempt to differentiate between weaker and stronger binders. Intermediate washing between each elution step with the same buffers yielded negligible binder erosion, as estimated by microbiological tittering of washes by transducing *E. coli* TG1 cells (Supplementary Fig. S1). This could indicate near-quantitative selective elution of binders released at specific pH, but may also be attributed to the binding kinetics (i.e., slow dissociation rate), since the washing steps were brief compared to the 8-min elution periods.

### Sequencing data analysis and hit ranking

The number of relevant NGS reads for the pre-screened library was 184,511, while the number of reads for individual eluates ranged from 5522 to 15,142 (median 10,898; all but one sample exciding 10,000 reads). The limited number of sequencing reads was a consequence of DNA sample preparation strategy; since pooled phagemids were fragmented, relatively few reads mapped to peptide-encoding inserts with vast majority mapping to the phagemid backbone. Nevertheless, the number of reads was sufficient to identify enriched clones with high confidence, as the library diversity was moderate. Targeted NGS relying on PCR amplification of insert regions was considered suboptimal since it might lead to introduction of sequence errors.

Residues enriched at individual randomized positions with regard to employed elution conditions are summarized in Table 1 (the relative enrichment factors for all the residues are shown in Supplementary Fig. S2). At position 2, histidine was the most enriched residue in peptides eluted at pH 2.2, 5.6, and 9.0, while amide group-containing residue asparagine was most prevalent in peptides eluted at pH 3.6. At positions 3 and 5, tyrosine was preferred over phenylalanine, although position 5 showed more tolerance for phenylalanine. Position 6 was most commonly occupied by asparagine and glutamine, the preference was most pronounced in peptides, eluted at pH 3.6 and 9.0. Two branched amino acid residues valine and isoleucine together with glutamine were strongly enriched at position 7, while position 9 showed a strong preference for phenylalanine over tyrosine. It should be noted that whenever glutamines were enriched, this can be largely attributed to the frequently occurring *amber* codon. However, since the *amber* suppression efficiency of the *E. coli* TG1 (*supE*) is estimated at 41–61%^23^, it is possible that the frequency of this residue was somewhat overestimated. Unexpectedly, we found that position 6 was somewhat biased against aspartate (most notably in elution step at pH 3.6 with 1.8-fold reduction; Supplementary Fig. S2)—i.e., the residue favored in the library based on our data from the previous study^10^.

**Table 1:** Relative enrichment factors for amino acid residues at individual randomized positions in peptides gathered at five elution conditions. The enrichment factors were calculated relative to the frequency of same residue at cognate position in pre-screened library.

Elution condition	pH 2.2		pH 3.6		pH 4.6		pH 5.6		pH 9.0	
Parallel experiment	A	B	A	B	A	B	A	B	A	B
**Position 2**										
D	1.28	1.24	1.34	1.24	1.17	1.28	1.28	1.10	1.55	1.24
H	5.45	4.75	3.65	3.10	2.45	2.50	4.95	5.50	5.40	5.25
N	2.85	2.35	5.05	5.25	2.30	2.35	1.80	1.80	3.30	3.05
Q	1.93	2.05	2.55	2.38	2.23	2.24	2.20	2.11	2.25	2.28
S	1.74	1.54	2.36	2.29	1.51	1.47	1.30	1.39	1.90	1.95
**Position 3**										
Y	1.79	1.60	2.26	2.16	1.83	1.76	1.72	1.79	2.14	2.04
**Position 5**										
Y	1.28	1.18	1.78	1.69	1.32	1.29	1.25	1.32	1.45	1.46
**Position 6**										
H	1.27	1.11	1.89	1.82	1.49	1.24	1.15	1.13	1.36	1.29
N	3.98	2.82	6.98	6.61	4.57	4.11	3.14	3.43	5.05	4.86
Q	3.45	2.75	4.37	4.70	2.35	2.61	2.29	2.20	3.61	3.61
S	1.50	1.36	2.25	1.86	1.79	1.68	1.29	1.36	2.00	1.64
**Position 7**										
I	1.73	1.44	1.73	1.62	1.56	1.51	1.47	1.56	1.84	1.82
Q	1.89	2.29	1.66	1.85	2.36	2.14	2.20	2.56	1.95	1.91
V	3.63	2.96	5.40	5.09	3.30	3.21	2.88	2.87	4.22	4.04
**Position 9**										
F	1.21	1.18	1.33	1.31	1.24	1.23	1.21	1.22	1.29	1.27

**Figure 3:**
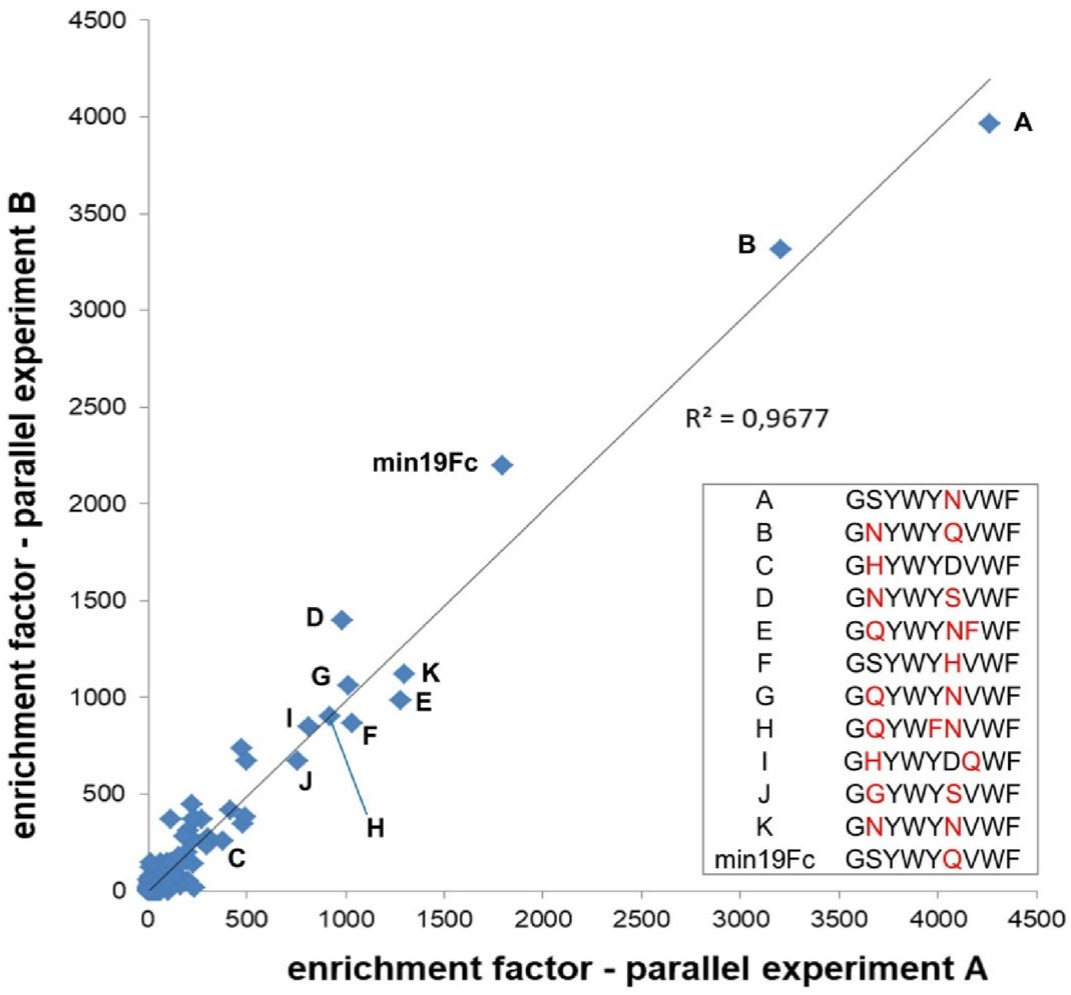
Correlation of enrichment factors for peptides eluted at pH 3.6 in 2 parallel experiments (N = 5090; each peptide detected at least once in at least one of the experiments). Peptide names are indicated in capital letters and their corresponding amino acid sequences are shown in the inset box. Residues differing from the parent peptide min19Fc-Q6D are shown in red.

Although a good indicator of library screening effectiveness and repeatability, the enrichment factors for individual residues at given positions are of limited value when it comes to selecting the optimal hits. Indeed, it might be that cooperative interactions evolved within the randomized clusters of residues, which account for the improved ligand binding properties, and these might not necessarily be recapitulated by a peptide having the consensus sequence. We thus calculated the enrichment factors for all 19,035 individual peptides eluted at 5 different conditions and ranked the hits accordingly (top 20 enriched peptides per elution condition are shown in Supplementary Table S1). In general, there was a high correlation between the enrichment factors of peptides from parallel experiments (Fig. 3 and Supplementary Fig. S3). The most strongly enriched peptide found in all eluates was GSYWYNVWF (termed peptide A) with enrichment factors ranging from ≈ 750 (at pH 5.6) to ≈ 4100 (at pH 3.6).

### Comparison of peptide affinities by phage ELISA

**Figure 4:**
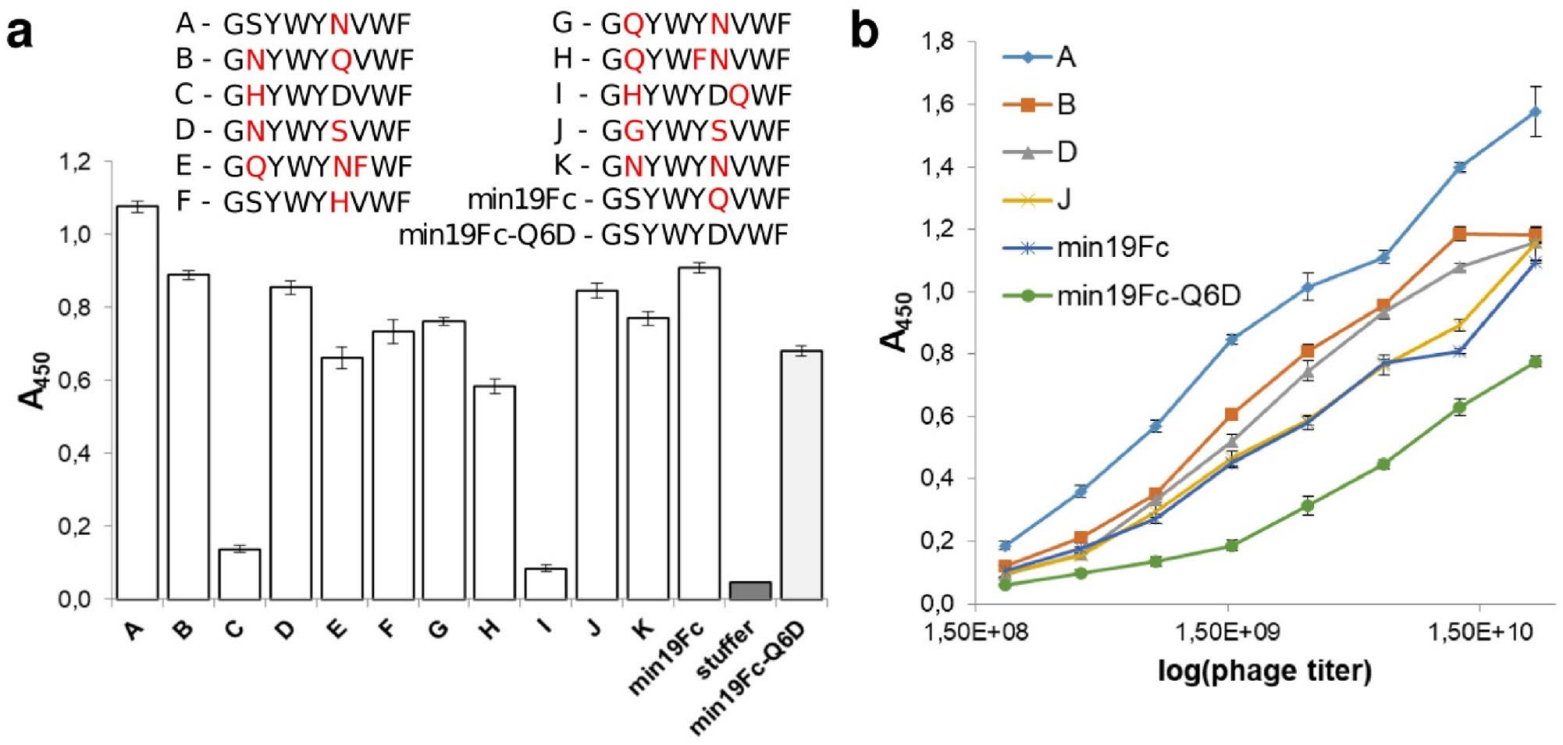
Assessment of relative affinities for human IgG of highly enriched clones. (**a**) Phage ELISA screen conducted with 2 × 10^9^ phage particles per well. (**b**) Phage ELISA assay conducted with two-fold dilutions of phage clones that gave highest signals in (**a**) Average values and standard deviations of 3 parallel experiments are presented.

As the same set of binders was enriched to various degrees across all elution conditions, we assessed the relative affinities of 12 phage-displayed peptides (denoted A to K, and min19Fc; occurring at highest frequencies) to human IgG pool with an ELISA assay in comparison to the parent peptide min19Fc-Q6D. The initial screen conducted with a single phage titer (Fig. 4a) suggested that peptide A displayed the highest affinity for IgG, followed by peptides B, D, J, and min19Fc. Interestingly, peptides C and I, both harboring histidine residue at position 2 and aspartate residue at position 6, although being consistently found among the top 20 highly enriched peptides, showed poor binding to IgG. Comparison of binding strengths for peptides G and H, which only differ in the aromatic residue at position 5 (tyrosine vs. phenylalanine, respectively), indicates that Tyr5 augments affinity, consistent with the preference for this residue in the consensus peptide (Table 1). A similar conclusion can be drawn from binding data for peptides E and G, where valine at position 7 is clearly superior to the bulky phenylalanine. Furthermore, binding data for peptides A, G, and K single out serine at position 2 as superior to amide group-containing residues asparagine and glutamine. Comparison of relative affinities for peptides A and F, and B and K, respectively, indicates that asparagine at position 6 is favorable for binding to IgG.

Next, we analyzed IgG binding of peptides A, B, D, J, and min19Fc at increasing titers (Fig. 4b). The binding curves corroborated well with the data collected at a single phage titer. Peptide A showed the strongest binding activity with estimated affinity two- to fourfold higher compared to peptides B, D, J, and min19Fc, and ≈ 16-fold higher compared to the parent peptide min19Fc-Q6D. Based on these data, peptide A was chosen as the ligand for the construction of chromatographic affinity matrix.

### Affinity column construction and characterization

Synthetic peptides A and min19Fc-Q6D were coupled to WorkBeads 40/1000 ACT resin funcionalized with high density bromohydrin reactive groups and pores large enough to accommodate IgGs. The coupling was performed in three steps. First, tris(2-aminoethyl)amine was reacted with the bromohydrin resin to introduce a branched linker that had previously been shown to increase the peptide coupling density, thereby enhancing binding capacity^17,24^. Next, the amine groups were acylated with bromoacetic acid to introduce sulfhydryl-reactive bromoacetyl groups. Finally, the peptides were coupled to the resin via the C-terminal cysteine residues. The coupling extent of 20–25 mg peptide per mL of resin was determined by measuring the 280 nm absorbances of the peptide solution before coupling and the supernatant after coupling. For a side-by-side functional comparison of the two peptide-based affinity matrices, we constructed 1 mL affinity columns. In addition, the functionalities of in-house affinity matrices were compared to the commercial spA-based affinity column (1 mL BabyBio A, Bio-Works) based on the same bromohydrin-activated agarose resin.

Initially, we performed test runs on the two in-house affinity columns to assess the optimal elution conditions. Since the peptide ligands described herein are hydrophobic, we assumed that the main driver of the IgG affinity are hydrophobic interactions. Elution at low pH of phage-displayed peptides during library screening is likely due to the (partial) antibody target denaturation. On the other hand, elution conditions need to be optimized in order to preserve the antibody integrity. The initial attempts to elute the bound antibodies with 200 mM glycine∙HCl buffer (pH 2.2) were only partially effective, and we noted that subsequent flushing of the column with water released most of the remaining adsorbed material. This indicated that low ionic strength buffer should be used for quantitative desorption. Indeed, we achieved highly efficient elution with 20 mM glycine∙HCl buffer (pH 3.0). We regularly performed CIP runs between chromatographic experiments and observed that even long exposure (several hours) to 0.5 M NaOH did not affect the columns’ functionality.

**Figure 5:**
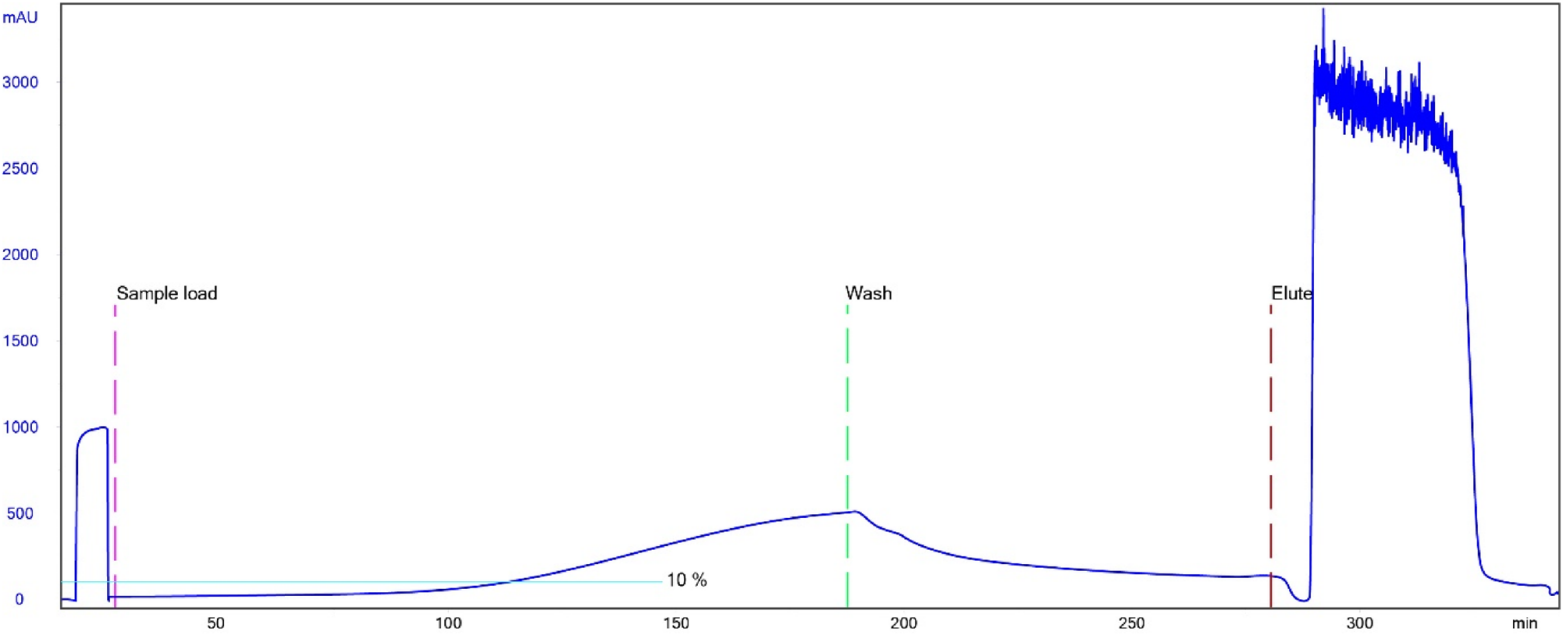
Chromatogram of DBC determination for peptide A 1 mL affinity column.

Next, DBC was assessed for all three columns at 10% breakthrough (Fig. 5 and Supplementary Figs. S4 and S5). Based on the flow rate (0.5 mL/min) and 10% breakthrough times, we calculated DBCs for peptide A-, min16Fc-Q6D-based and BabyBio A columns to be 42.9 mg/mL, 22.7 mg/mL and 41.3 mg/mL, respectively, for infliximab (an IgG1 chimeric antibody). This means that the peptide A-based affinity column showed approximately twofold higher DBC compared to the min19Fc-Q6D-based one, and that DBC of peptide A matrix is on par with that of BabyBio A. This was further confirmed by the amount of infliximab in individual eluates (43.7 mg, 9.9 mg, and 41.2 mg for peptide A, min19Fc-Q6D and BabyBio A column, respectively, as determined spectrophotometrically). Since the eluted amount of antibody from the min19Fc-Q6D column is lower than expected from the calculated DBC, we assume that a significant proportion of it was lost during the washing step. This is corroborated by the shape of the chromatogram upon washing (Supplementary Fig. S4) and can be attributed to the lower affinity of min19Fc-Q6D for IgG as compared to peptide A.

**Figure 6:**
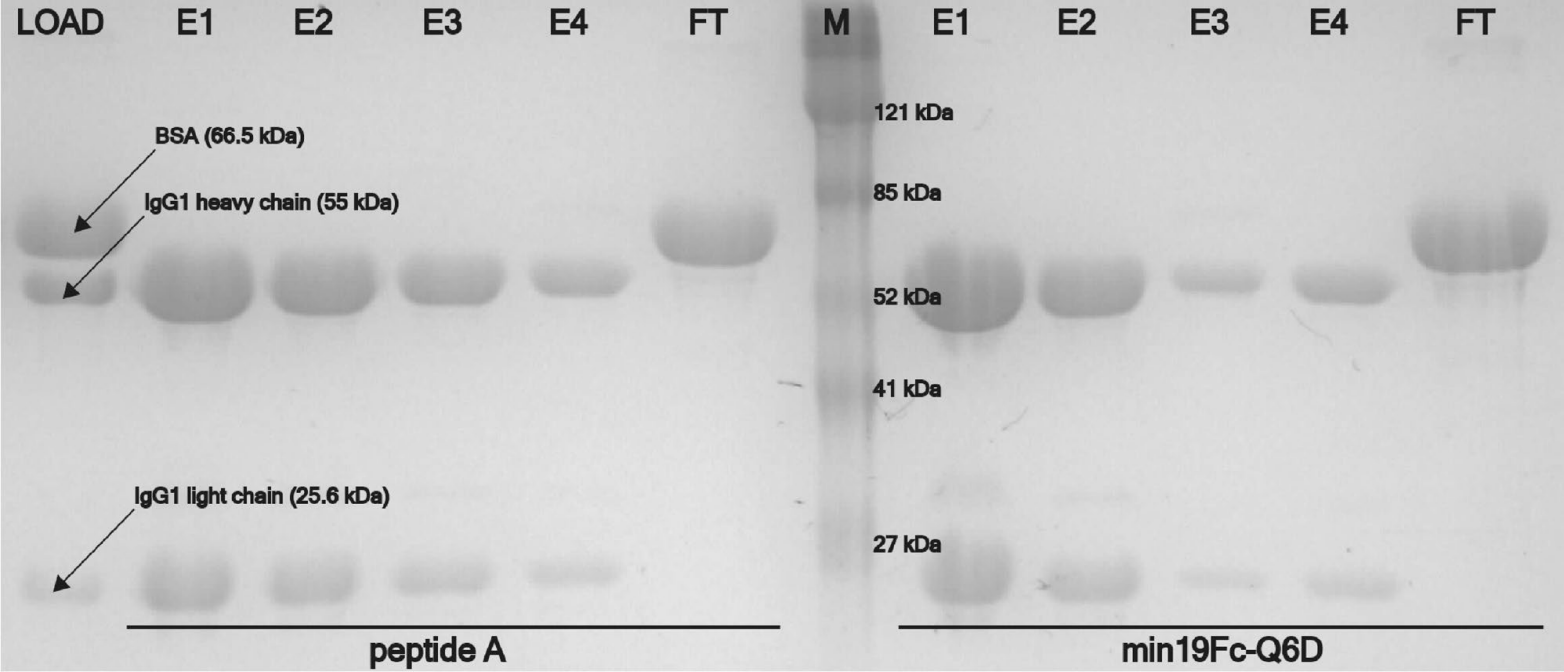
SDS-PAGE analysis of eluted fractions for assessment of binding specificity. A 1:4 mixture (mass ratio) of infliximab (chimeric IgG1) and BSA was loaded on the columns. E1–E4—sequential 1.5 mL elution fractions; FT—low-through; M—protein marker (ProSieve color protein marker, Lonza).

The columns were evaluated for binding specificity. Infliximab was mixed with BSA at 1:4 mass ratio and the mixture was loaded onto the columns (Supplementary Figs. S6–S8). All three columns showed high specificity with no BSA present in the eluate as analyzed by SDS-PAGE (Fig. 6 and Supplementary Fig. S9).

**Figure 7:**
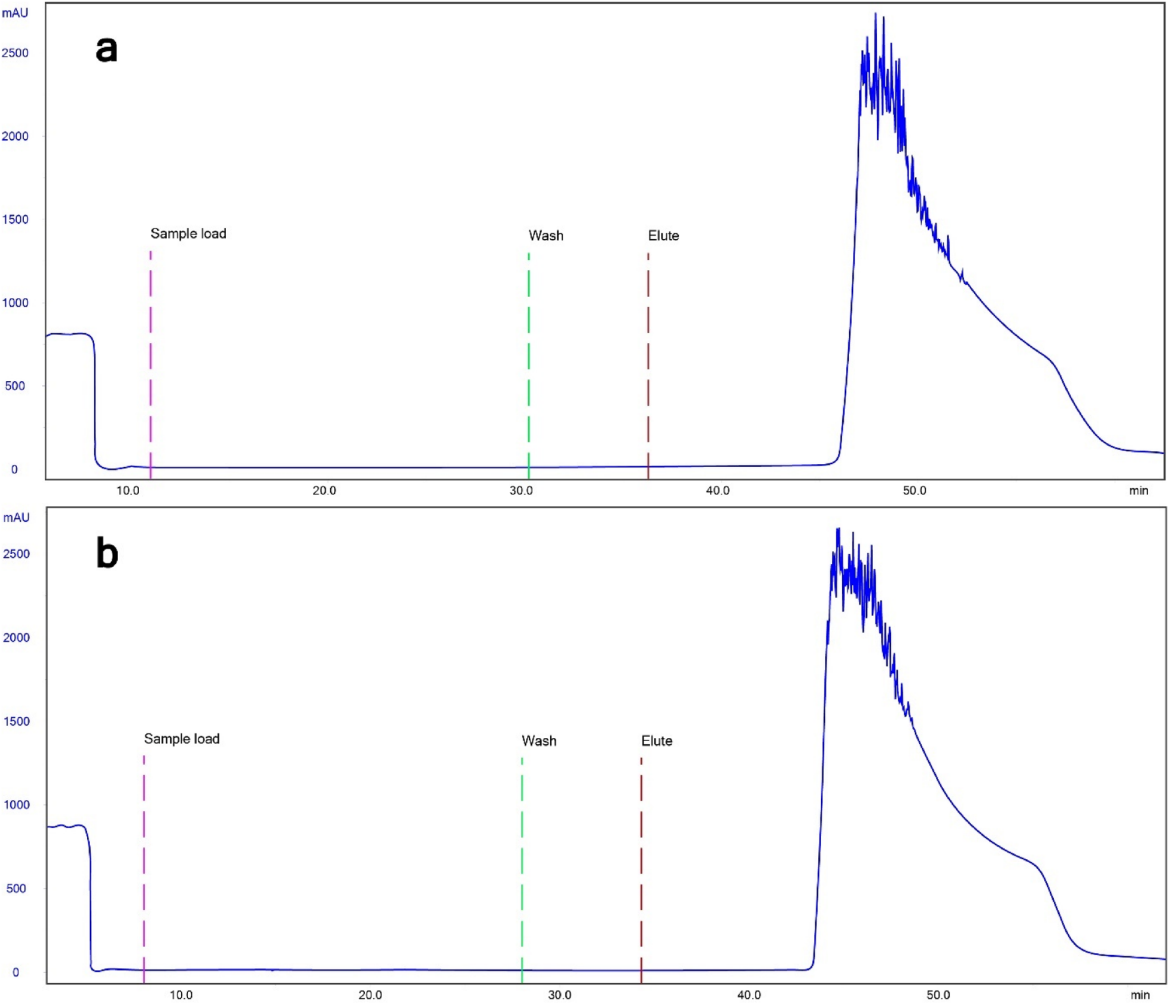
Chromatograms showing adsorption and elution of panitumumab (IgG2; **a**) and nivolumab (IgG4; **b**) for peptide A-based affinity column.

Lastly, we have tested the peptide A-based column for binding IgG2 (panitumumab; 5 mg) and IgG4 (nivolumab; 6 mg; Fig. 7). Since these were only used in small quantities, breakthrough upon loading was not reached, and elution peak shapes differ from that of the chromatogram in Fig. 5. Figure 7 thus depicts results of qualitative analyses. A similar chromatogram was recorded upon loading 1 mg of polyclonal human IgG3 (Supplementary Fig. S10). These data show that the peptide A-based affinity matrix is capable of reversibly adsorbing all four human IgG subclasses.

## Discussion

High titers achieved by recombinant expression in mammalian cells have shifted the majority of antibody production costs to downstream processing. While affinity chromatography based on bacterial immunoglobulin-binding proteins, such as staphylococcal protein A, still represents the cornerstone of antibody isolation and purification, it is associated with high operational costs due to ligand instability. Moreover, high affinity of protein A to IgG requires rather harsh elution conditions that may be detrimental to both, the matrix-coupled ligand and the antibody product. Hence, robust adsorption matrices based on alternative affinity ligands providing uncompromised selectivity for antibodies are sought after^1,6,25^. The combinatorial nature of peptides provides unprecedented diversity, forming the basis for high affinity/high selectivity binders. It also allows for applying the principles of molecular evolution to identify and affinity maturate peptide ligands. This is accomplished by constructing libraries of peptide variants based on synthetic approaches and exerting selection pressure to enrich members displaying the desired property. Here, we report affinity maturation of a peptide binding to the Fc region of IgG by screening a phage display library. Further, we demonstrate that an agarose support functionalized by the synthetic optimized peptide has functional properties comparable to those of a commercial protein A-based matrix.

By randomizing the residues of the parent peptide min19Fc-Q6D at specific positions that were observed to tolerate substitutions without major loss of Fc region-binding affinity, we constructed a small focused phagemid-displayed library. The library was used to systematically probe the chemical space in an attempt to identify the optimal ligand from a set of peptides with the general structure GX[Y/F]W[Y/F]XXW[Y/F] (where X denotes any amino acid). Massive parallel sequencing of virions recovered after affinity selection provided a detailed insight into the enrichment of clones. The same set of peptides was enriched to various degrees in two parallel independent experiments regardless of the elution conditions (i.e., across all applied pH values). The peptide min19Fc-Q6D (GSYWYDVWF) which was considered to be an improved variant of the initially identified lead peptide min19Fc (GSYWYQVWF) and was used as the basis of the secondary library, was only moderately enriched (enrichment factors of approx. 12–25). On the other hand, the peptide min19Fc was consistently highly enriched (enrichment factors of approx. 440–1990). This was unexpected since all previous data pointed to the superiority of min19Fc-Q6D^10^. The reason for this is not clear, but might be linked to the different display mode (e.g. multivalent vs. monovalent display, and/or differences in linker flexibility and hydrophilicity/charge). Interestingly, a single homologous substitution at position 6 (asparagine in peptide A in place of glutamine in min19Fc) turned out to significantly augment the ligand’s affinity for Fc region. Under all elution conditions the peptide A was highly enriched (enrichment factors of approx. 750–4100; Supplementary Table S1) and displayed highest affinity for polyclonal human IgG among all tested peptides, as assessed in phage ELISA assay (Fig. 4). Using the same assay, we also checked whether peptide A and min19Fc-Q6D bind murine, goat and donkey IgGs. No interaction was detected, indicating that both peptides are specific for human IgGs (not shown).

Both, the peptide min19Fc-Q6D, parent to the secondary library, and the peptide A were coupled to activated agarose matrix via a short-branched linker and evaluated for IgG binding in comparison to commercial affinity matrix based on staphylococcal protein A (BabyBio A). Binding/washing buffer was supplemented with a low concentration (0.01%) Tween-20 to stabilize the antibodies and limit their aggregation. Low ionic strength (20 mM glycine∙HCl) was found to be required for IgG desorption, while the pH of elution buffer was chosen to be the same as recommended for the protein A affinity column (pH 3.0). Under these conditions, peptide A-functionalized resin quantitatively adsorbed infliximab (IgG1) with DBC comparable to BabyBio A (approx. 40 mg/mL resin; Fig. 5). Elution peak was, however, indicative of prolonged elution, likely due to the slow drop of ionic strength upon washing and elution buffer mixing on the column. Affinity column based on the peptide min19Fc-Q6D displayed a twofold lower apparent DBC. However, a significant proportion of adsorbed antibodies were lost during washing at an increased flow rate as judged from quantification of eluted material (contrary to peptide A-based column), in good agreement with the lower affinity. In contrast to protein A, peptide A reversibly bound all human IgG subclasses (i.e., even IgG3) and demonstrated high tolerance to alkaline conditions (several hours at 0.5 M NaOH).

Several other peptide-based ligands for IgG affinity chromatography have been reported^17,26–30^, but a direct comparison of the corresponding affinity resins to our matrix is difficult due to the use of different stationary phases along with differences in ligand functional densities (in some cases dendrimers were used), coupling linkers, and/or diverse chromatographic conditions. Our peptide affinity matrix best compares to the ones constructed on WorkBeads support by Lund et al.^31^ (utilizing the ligand 2,6-di-t-butyl-4-hydroxybenzyl-Arg-Arg-Gly, termed DAAG)) and Islam et al.^17^ (functionalized with the peptide HWRGWV using the same triamine linker as in our case). DBCs of all three fall in the similar range (≈ 40–60 mg IgG/mL resin) at approximately the same ligand density.

## Conclusions

Conventional protein A chromatography is widely applied for the isolation and purification of antibodies, yet it is associated with high operational costs. In the current report, we describe the development of an alternative affinity chromatography based on the short linear synthetic peptide GSYWYNVWFC. The structure (and hence affinity) of the peptide ligand was optimized through focused combinatorial phagemid-based library screening and massive parallel sequencing to identify the highly enriched clones. When coupled to an agarose matrix via a short branched linker, the peptide ligand enabled selective adsorption of all human IgG subclasses, and the affinity matrix displayed high resistance to alkaline treatment (typically applied during column decontamination).

Although the results of the current study are auspicious, several lines of research need to be pursued in order to better characterize and further optimize the affinity column. Specifically, our current efforts are directed at understanding the molecular interactions between the IgG Fc region and the peptide ligand, setting the basis for improvement of chromatographic conditions (particularly the elution step). In the reported proof-of-principle chromatographic experiments we have shown that low ionic strength of the elution buffer allows for quantitative IgG desorption at pH 3.0, which is at the high end of typical protein A-based affinity columns. Furthermore, long-term stability of the affinity matrix (i.e., monitoring the potential decline in DBC over time) will need to be thoroughly analyzed, and potential selectivity of the peptide A-functionalized matrix for the monomeric IgG form over aggregates (as has been reported for several other mixed-mode^32^ and peptide-based resins^17,31^) warrants checking. Additionally, the performance of the affinity matrix upon complex feedstock loading will need to be evaluated, as well as its resistance to proteases and diverse cleaning-in-place conditions. Aside from affinity chromatography, peptides described herein might find use in other applications, such as ligands for homogenous immobilization of detection antibodies on biosensor surfaces, or capture antibodies on immunoprecipitation beads, provided the binding affinity is appropriately high.

## Supplementary Information

Supplementary Information.

## Data Availability

The modified phagemid pIT2-SL and its nucleotide sequence are available on request. Requests should be placed to the corresponding author’s address or email.
