# Supplementary material for: Focused peptide library screening as a route to a superior affinity ligand for antibody purification
Source: Sci Rep. 2021 Jun 2;11:11650. doi: 10.1038/s41598-021-91208-0 (PMC8173005; doi:10.1038/s41598-021-91208-0)
Supplement: Supplementary file 1 — Supplementary Information. [file 41598_2021_91208_MOESM1_ESM.pdf]

Supplementary information

Focused peptide library screening as a route to a superior affinity ligand for antibody purification

Krištof Bozovičar[1], Barbara Jenko Bizjan[2], Anže Meden[3], Jernej Kovač[2], Tomaž Bratkovič[1,*]

[1] University of Ljubljana, Faculty of Pharmacy, Department of Pharmaceutical Biology, Aškerčeva 7, SI-1000 Ljubljana, Slovenia
[2] University Medical Center, University Children's Hospital, Clinical Institute of Special Laboratory Diagnostics, Vrazov trg 1, Ljubljana, Slovenia
[3] University of Ljubljana, Faculty of Pharmacy, Department of Pharmaceutical Chemistry, Aškerčeva 7, SI-1000 Ljubljana, Slovenia

* Correspondence: Tomaž Bratkovič (tomaz.bratkovic@ffa.uni-lj.si)

Supplementary figures

Supplementary Figure S1: Plating of eluted phagemid virion-transduced host bacteria on selection LB-agar (containing 100 µg/mL ampicillin). Top row: 50 µL (6.25%) of 1000-fold diluted eluates collected at pH 5.6, 4.6, 3.6, and 2.2 (left to right) were used to infect *E. coli* TG1 and plated. Bottom row: 50 µL (1.25%) of undiluted respective washes following elution step were used to infect *E. coli* TG1 and plated.

Supplementary Figure S2: Fold enrichment (relative to the residue frequency in pre-screened phage library) per individual randomized positions for different elution conditions.

Supplementary Figure S3: Correlation of enrichment factors for peptides eluted at different pH conditions in 2 parallel experiments.

Supplementary Figure S4: Chromatogram of DBC determination for min19Fc-Q6D 1 mL affinity column.

Supplementary Figure S5: Chromatogram of DBC determination for BabyBio A 1 mL affinity column.

Supplementary Figure S6: Chromatogram of specificity assessment for peptide A column.

Supplementary Figure S7: Chromatogram of specificity assessment for min19Fc-Q6D column.

Supplementary Figure S8: Chromatogram of specificity assessment for BabyBio column.

Supplementary Figure S9: SDS-PAGE analysis of eluted material for assessment of binding specificity for BabyBio column. E – eluted fraction; FT – low-through; M – protein marker (ProSieve color protein marker, Lonza).

Supplementary Figure S10: Chromatogram showing adsorption and elution of polyclonal human IgG3 for peptide A-based affinity column.

Supplementary table

Supplementary Table S1: Relative enrichment factors (EF) for the top 20 peptides gathered at five elution conditions. Enrichment factors were calculated relative to the frequency of same peptide in pre-screened library. Peptide A (GSYWYNVWF) highly enriched in all eluates is shown shaded dark grey, while the peptide min19Fc (GSYWYQVWF), a minimized variant of a ligand discovered by screening a random peptide library[1], is shaded light grey. peptide B – blue; peptide C – violet; peptide D – magenta; peptide E – red; peptide F – grey; peptide G – green; peptide H – dark orange; peptide I – olive green; peptide J – light orange; peptide K – cian.

Supplementary Figure S1

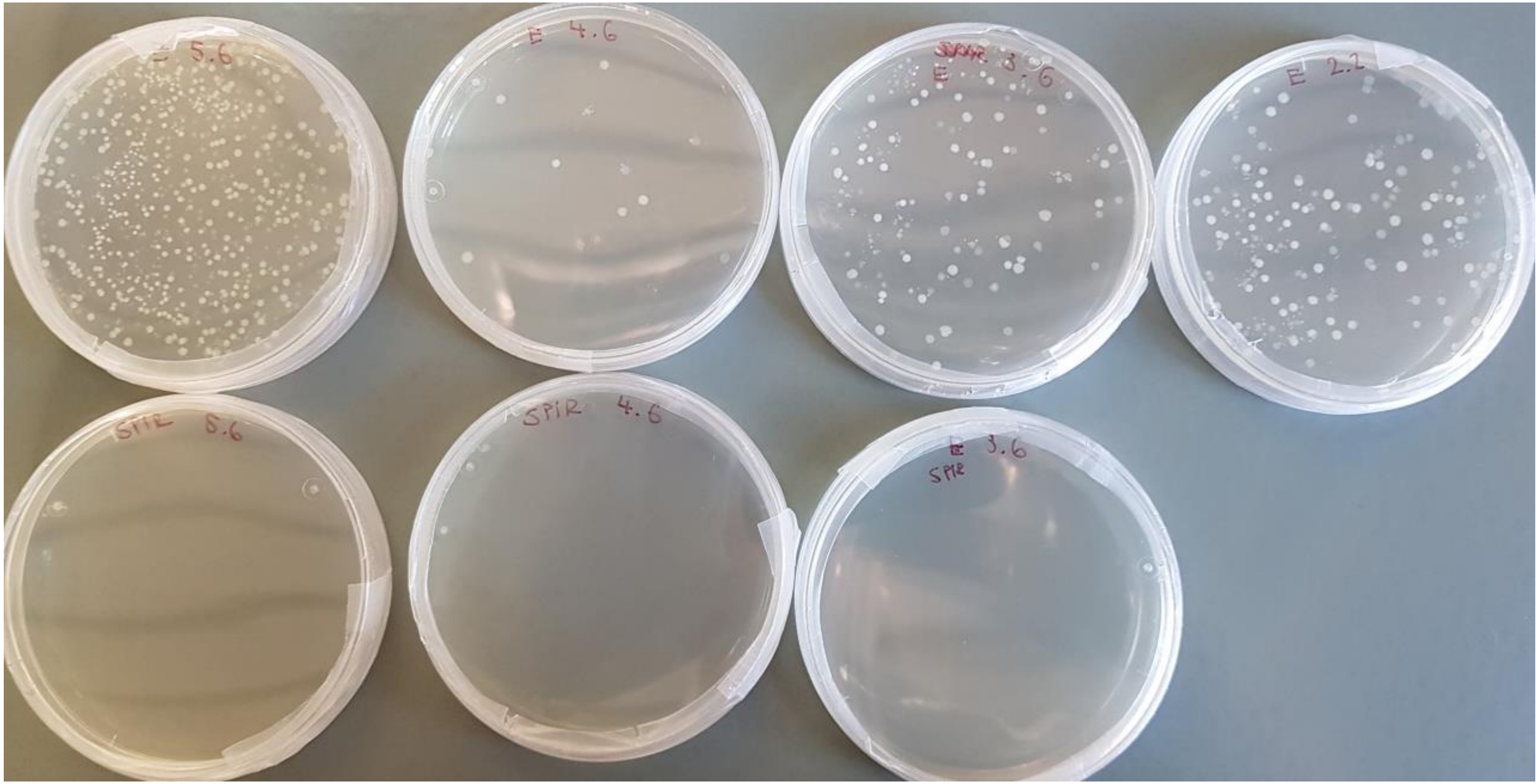

Supplementary Figure S2

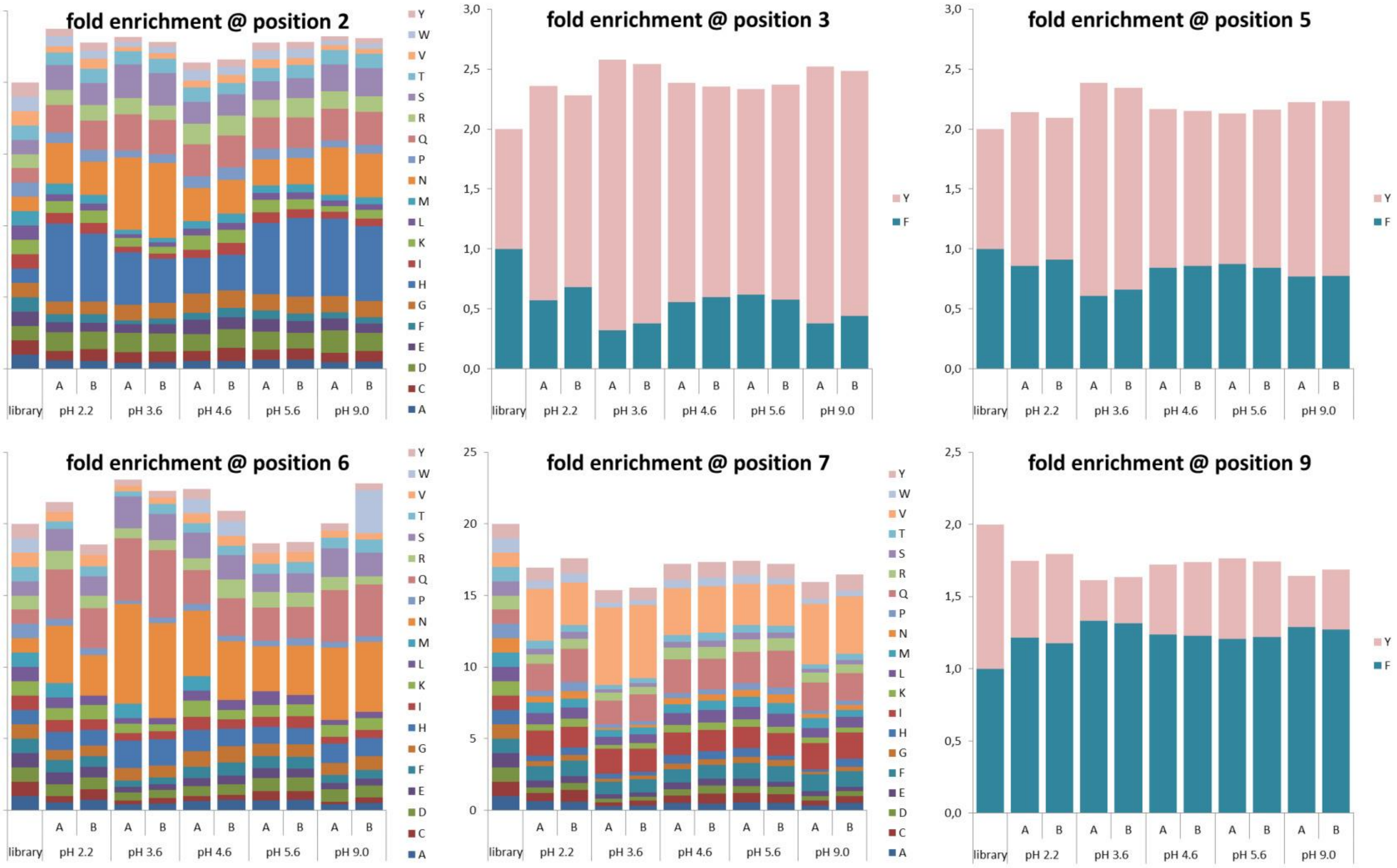

Supplementary Figure S3

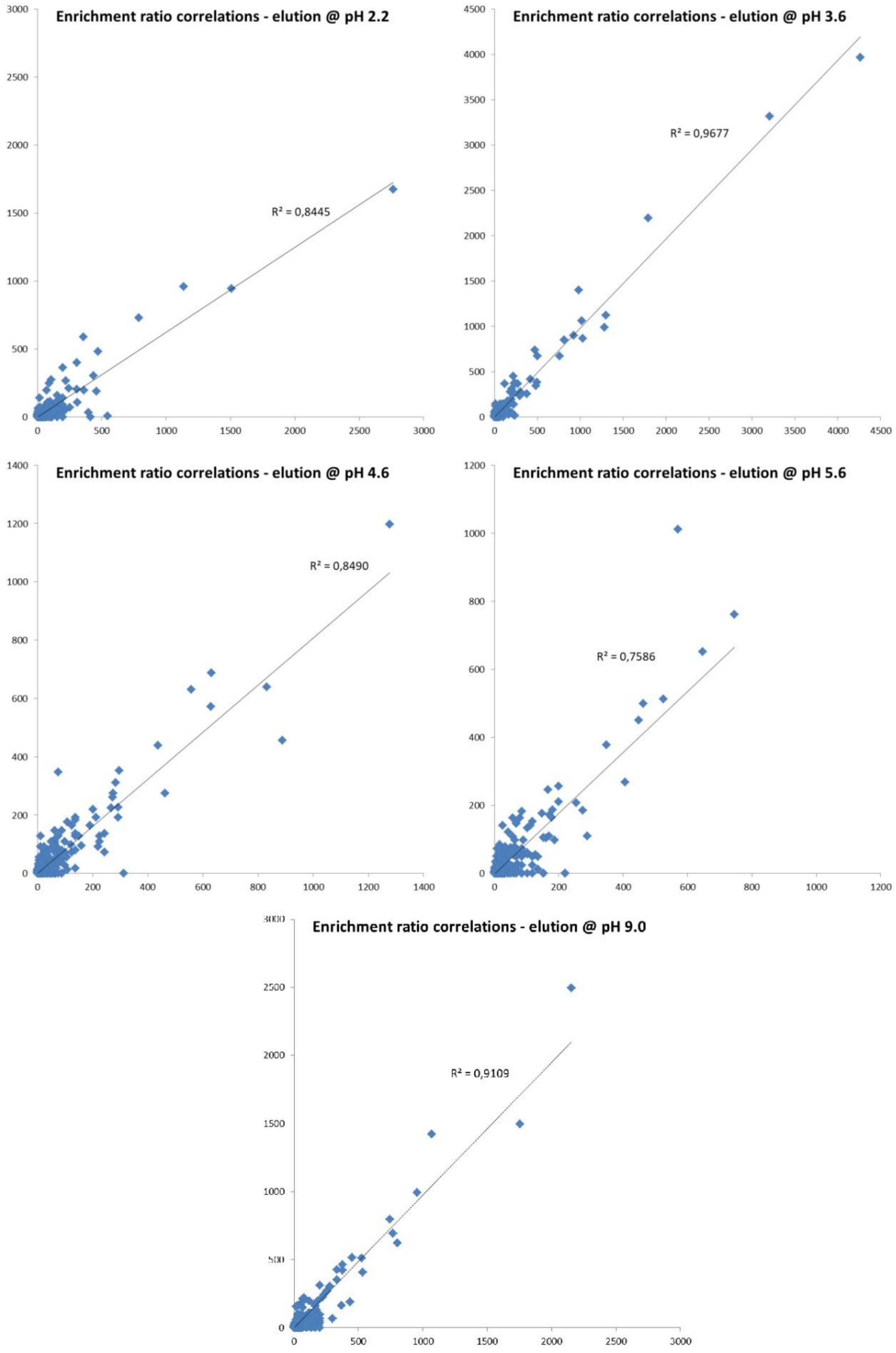

Supplementary Figure S4

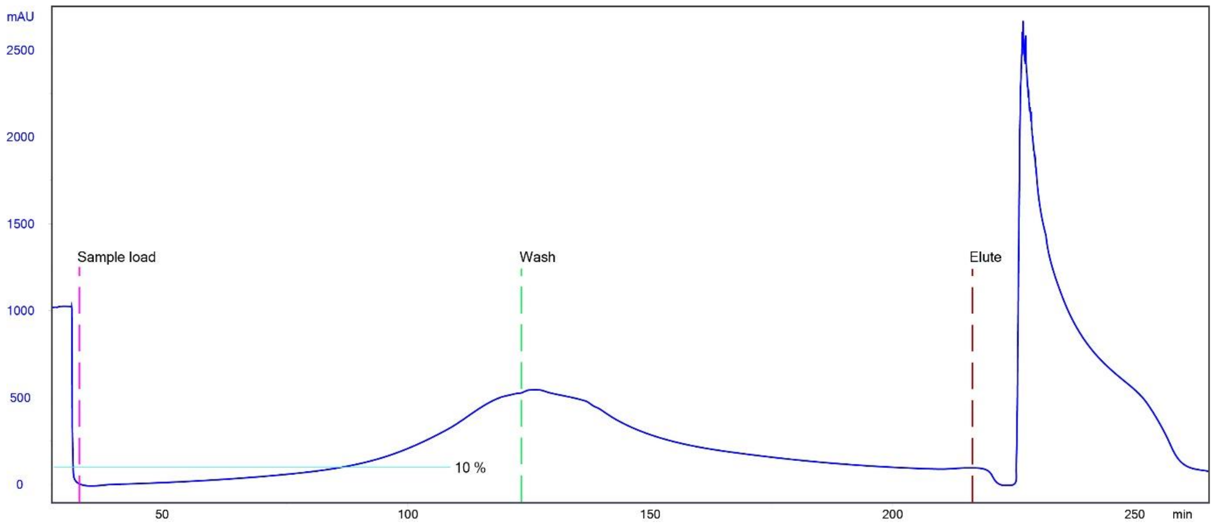

Supplementary Figure S5

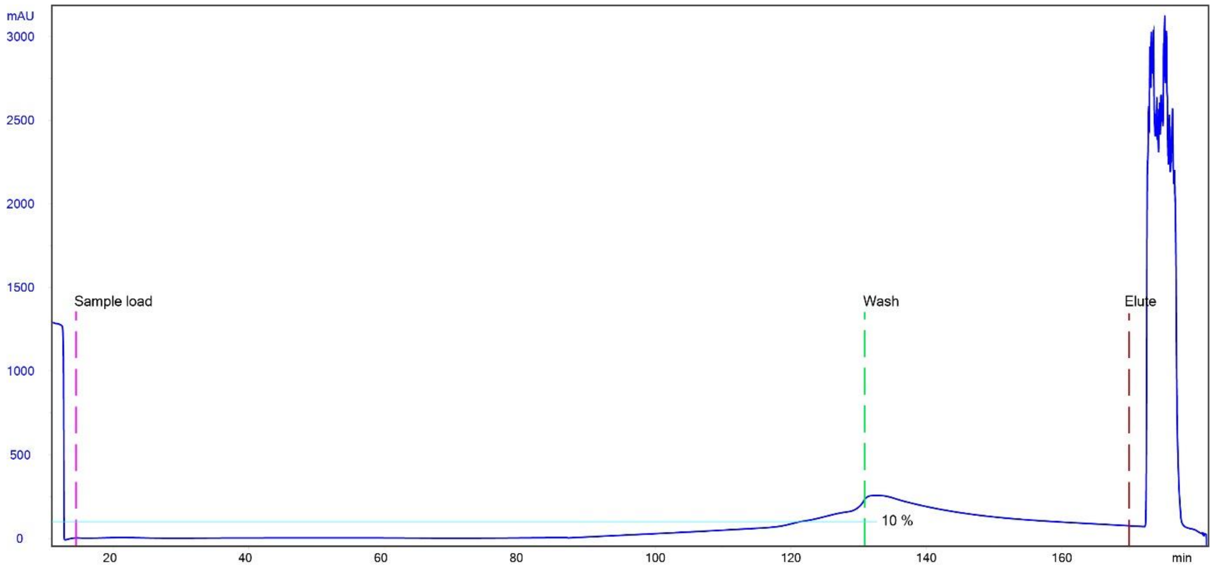

Supplementary Figure S6

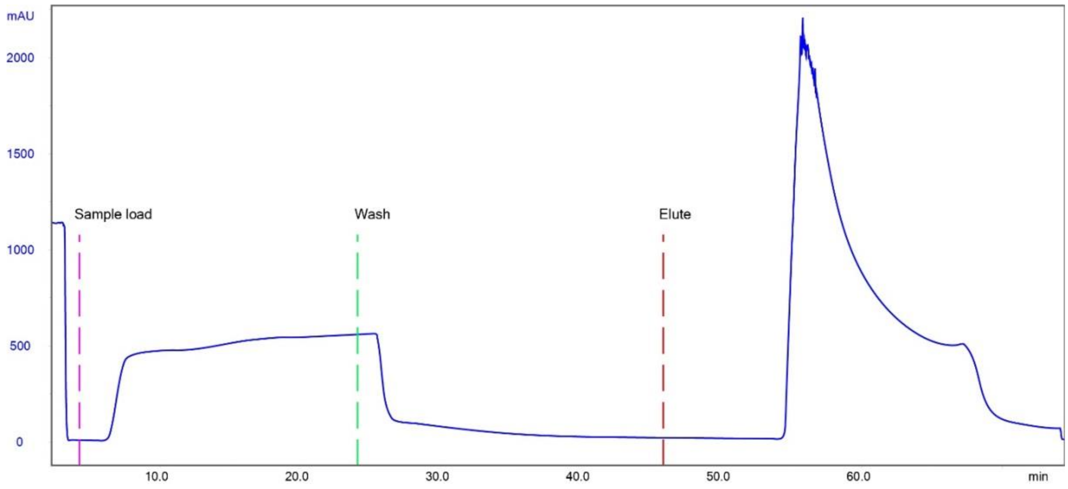

Supplementary Figure S7

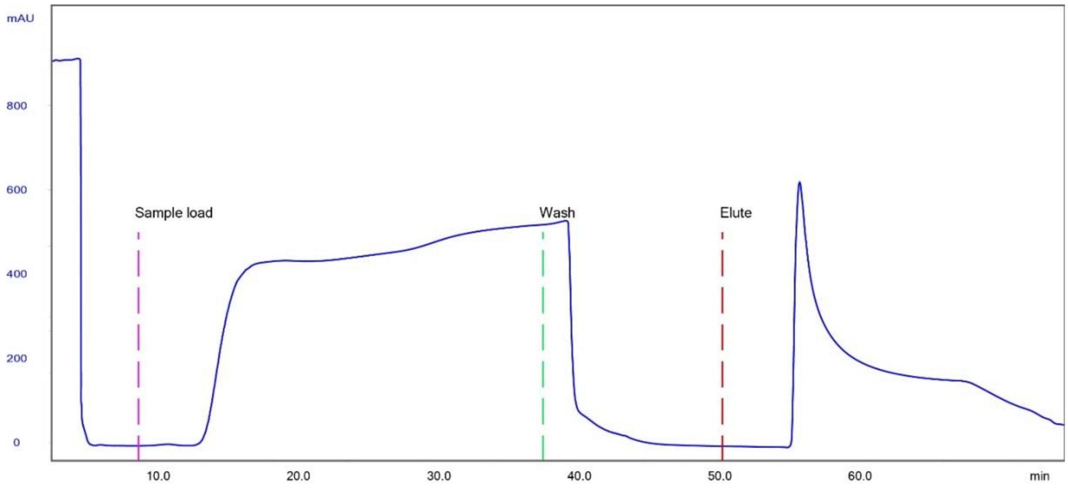

Supplementary Figure S8

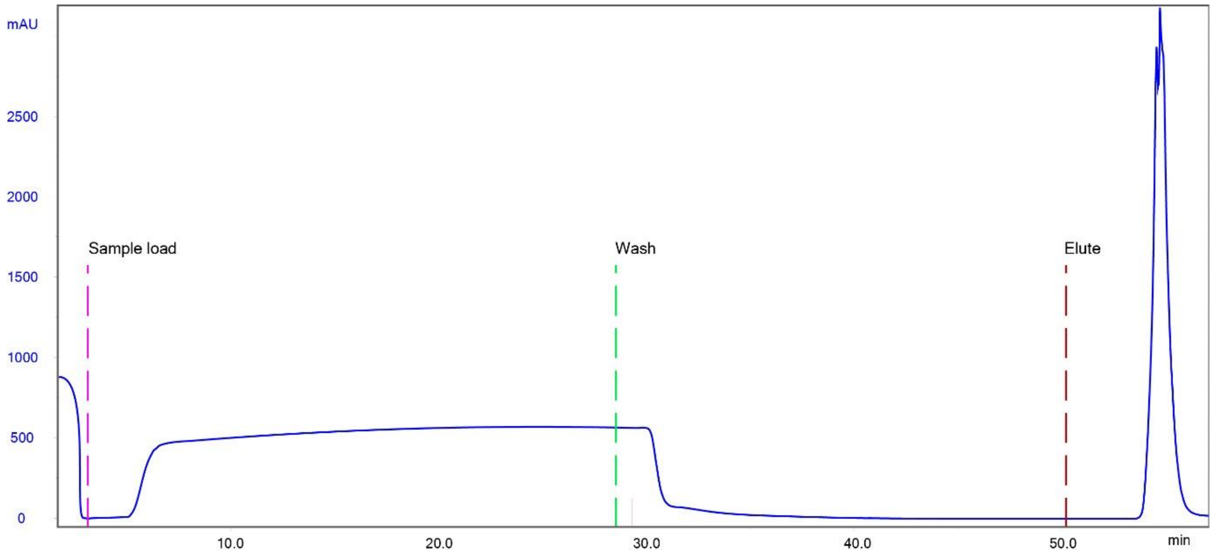

Supplementary Figure S9

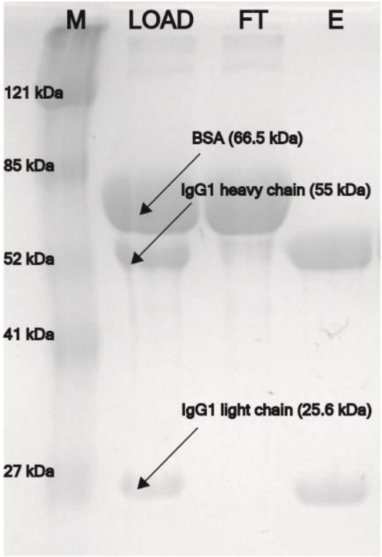

Supplementary Figure S10

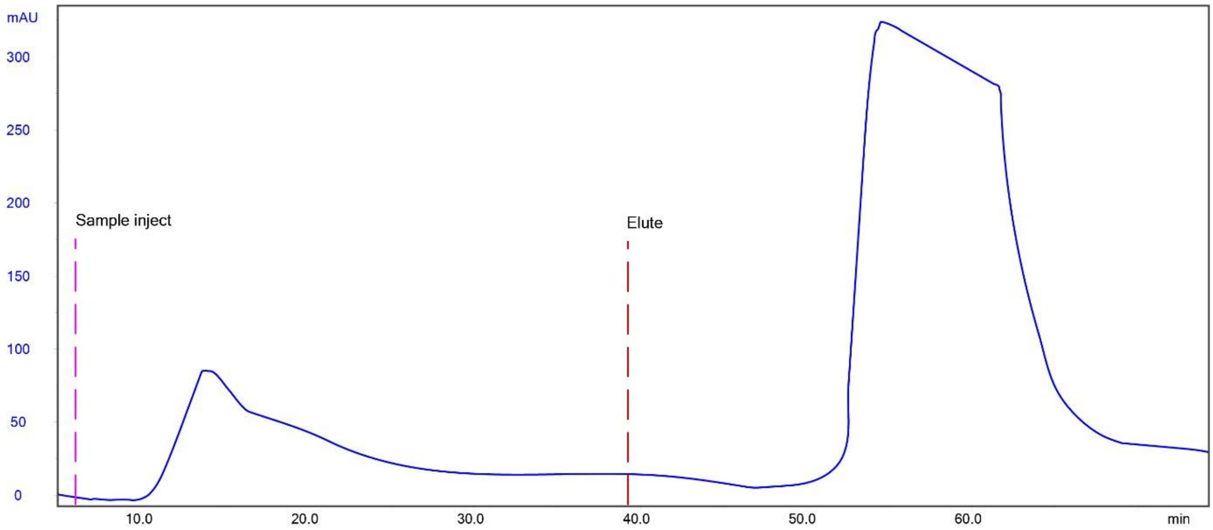

Supplementary Table S1

	elution @ pH 2.2				elution @ pH 3.6				elution @ pH 4.6				elution @ pH 5.6				elution @ pH 9.0			
	parallel experiment A		parallel experiment B		parallel experiment A		parallel experiment B		parallel experiment A		parallel experiment B		parallel experiment A		parallel experiment B		parallel experiment A		parallel experiment B	
**rank**	**peptide**	**EF**	**peptide**	**EF**	**peptide**	**EF**	**peptide**	**EF**	**peptide**	**EF**	**peptide**	**EF**	**peptide**	**EF**	**peptide**	**EF**	**peptide**	**EF**	**peptide**	**EF**
1	**GSYWYNVWF**	2764	**GSYWYNVWF**	1675	**GSYWYNVWF**	4263	**GSYWYNVWF**	3963	**GSYWYNVWF**	1276	**GSYWYNVWF**	1197	**GSYWYNVWF**	745	GHYWYDQWF	1011	**GSYWYNVWF**	2155	**GSYWYNVWF**	2495
2	GNYWYQVWF	1507	GHYWYDVWF	959	GNYWYQVWF	3206	GNYWYQVWF	3315	GNYWYSVWF	889	GQYWYNFWF	689	GHYWYDVWF	647	**GSYWYNVWF**	762	GNYWYQVWF	1754	GNYWYQVWF	1497
3	GHYWYDVWF	1137	GNYWYQVWF	944	**GSYWYQVWF**	1793	**GSYWYQVWF**	2195	GNYWYQVWF	832	GNYWYQVWF	640	GHYWYDQWF	570	GHYWYDVWF	652	**GSYWYQVWF**	1069	**GSYWYQVWF**	1424
4	**GSYWYQVWF**	790	**GSYWYQVWF**	730	GNYWYNVWF	1298	GNYWYSVWF	1400	GQYWYNFWF	630	GQYWFNVWF	631	**GSYWYQVWF**	525	**GSYWYQVWF**	512	GQYWYNFWF	958	GQYWYNFWF	993
5	GGYWFSVWF	547	GNYWYSVWF	588	GQYWYNFWF	1280	GNYWYNVWF	1123	GGYWYSVWF	629	GGYWYSVWF	571	GQYWYNFWF	463	GQYWYNFWF	500	GNYWYSVWF	802	GHYWYDVWF	796
6	GNYWYNVWF	471	GNYWYNVWF	481	GSYWYHVWF	1032	GQYWYNVWF	1061	GQYWFNVWF	557	GNYWYSVWF	457	GNYWYQVWF	449	GNYWYQVWF	451	GHYWYDQWF	769	GHYWYDQWF	693
7	GQYWYNFWF	461	GQYWFNVWF	401	GQYWYNVWF	1015	GQYWYNFWF	987	GQYWYNVWF	463	**GSYWYQVWF**	439	GNYWYSVWF	407	GQYWFNVWF	378	GHYWYDVWF	746	GNYWYSVWF	622
8	GSYWYHVWF	437	GSYWYSVWF	362	GNYWYSVWF	981	GQYWFNVWF	902	**GSYWYQVWF**	438	GHYWYDQWF	353	GQYWFNVWF	347	GNYWYSVWF	268	GSYWYHIWF	535	GQYWFNVWF	515
9	GHYWYDIWF	362	GSYWYHVWF	306	GQYWFNVWF	922	GSYWYHVWF	867	GHFWFKQWF	313	GDYWFNVWF	347	GCYWYNIWF	288	GGYWYSVWF	256	GGYWYSVWF	526	GGYWYSVWF	511
10	GNYWYSVWF	359	GPYWFQVWF	273	GHYWYDQWF	812	GHYWYDQWF	850	GHYWYDQWF	296	GGYWYGVWF	311	GSYWYHVWF	274	GHYWYDIWF	246	GQYWFNVWF	451	GNYWYNVWF	462
11	GDYWFQVWF	311	GHYWYDQWF	267	GGYWYSVWF	757	GGYWYSVWF	670	GSYWYGVWF	293	GQYWYNVWF	274	GQYWYNVWF	254	GNYWYNVWF	210	GDYWYHVWF	434	GQYWYNVWF	425
12	GQYWFNVWF	305	GQYWFELWF	249	GSYWYHIWF	499	GSYWYHIWF	670	GSYWYHVWF	292	GCYWYNIWF	274	GGYWYSVWF	199	GQYWYNVWF	207	GSYWYHVWF	379	GSYWYHVWF	423
13	GSYWYHIWF	305	GHYWFDVWF	213	GSYWYSVWF	496	GQYWYNYWF	449	GGYWYGVWF	284	GNYWYNVWF	260	GNYWYNVWF	199	GGYWYGVWF	187	GNYWYNVWF	376	GSYWYHIWF	409
14	GQYWYNVWF	251	GSYWYHIWF	205	GDYWYHVWF	482	GSYWYGVWF	415	GCYWYNIWF	275	GSYWYGVWF	227	GHFWYQVWF	186	GSYWYHVWF	185	GQYWYNVWF	334	GQYWYNYWF	352
15	GHYWFDVWF	244	GHYWYDIWF	196	GSYWYGVWF	420	GSYWYSVWF	383	GNYWYNVWF	272	GGYWYNVWF	225	GGYWYGVWF	181	GQYWYNYWF	183	GQYWYNYWF	334	GDYWFNVWF	311
16	GHYWYDQWF	221	GQYWYNFWF	190	GHYWYDVWF	378	GGYWYGVWF	373	GGYWYNVWF	268	GHYWYDVWF	219	GSYWYHIWF	178	GGYWYNVWF	177	GHYWFYQWF	301	GSYWYSVWF	305
17	GSYWFHQWF	215	GGYWYSVWF	160	GGYWYNVWF	305	GNYWFNVWF	372	GGYWFHQWF	244	GRYWYSVWF	192	GHYWFDVWF	171	GHYWFDVWF	172	GSYWYSVWF	278	GSYWYGVWF	272
18	GDYWFRIWF	197	GDYWFRIWF	143	GRYWYNVWF	297	GAYWYHVWF	368	GGYWYNIWF	244	GSYWYHIWF	192	GDYWFNVWF	169	GSYWYHIWF	165	GSYWYGVWF	261	GGYWYNVWF	268
19	GNYWFTFWF	197	GSYWFNVWF	129	GDYWFNVWF	271	GDYWFNVWF	368	GRYWYNLWF	225	GSYWYHVWF	192	GHYWYDIWF	166	GSYWFNQWF	162	GGYWYNVWF	251	GGYWYGVWF	240
20	GSYWYSVWF	197	GQYWYNYWF	116	GGYWYGVWF	242	GDYWYHVWF	346	GSYWFHQWF	225	GSYWYSVWF	183	GGYWYNIWF	161	GSYWYNLWF	162	GGYWYGVWF	234	GHYWYDIWF	223

Reference

1. Kruljec, N., Molek, P., Hodnik, V., Anderluh, G. & Bratkovič, T. Development and characterization of peptide ligands of immunoglobulin G Fc region. Bioconjug. Chem. 29, 2763–2775 (2018).
